# Systemic Inflammatory Response Index Has a Value for the Early Diagnosis and Short‐Term Prognostic Assessment of Severe Pneumonia in the Elderly

**DOI:** 10.1155/carj/6968736

**Published:** 2026-01-06

**Authors:** Xiao Xu, Huajuan Xu, Ming Li, Shuying Yan, Huilin Chen

**Affiliations:** ^1^ Intensive Care Unit (ICU), Shanghai Jiangong Hospital, Shanghai, 200083, China

**Keywords:** early diagnosis, elderly, severe pneumonia, short-term prognosis, systemic inflammatory response index

## Abstract

This article probed the value of systemic inflammatory response index (SIRI) in the early diagnosis and short‐term prognostic assessment of severe pneumonia (SPM) in the elderly. First, 266 elderly patients with pneumonia were retrospectively included and allocated into SPM and N‐SPM groups as per APACHE‐II scores. Correlations of SIRI with APACHE‐II scores, CRP, and PCT were analyzed. Patients were followed up for 28 days, and SPM patients were allocated into death and survival groups based on survival status. The values of SIRI for early diagnosis and short‐term prognostic assessment of elderly SPM patients were evaluated. Influencing factors for short‐term death in elderly SPM patients were screened. SIRI levels were high in elderly SPM patients and were positively correlated with APACHE II scores and CRP and PCT levels. SIRI had a high predictive value for the occurrence (AUC = 0.843) and 28‐day survival (AUC = 0.806) of SPM. High SIRI levels elevated the risk of 28‐day mortality in elderly SPM patients. APACHE II scores (HR = 1.125) and SIRI (HR = 1.977) were independent risk factors for short‐term death in elderly SPM patients. Overall, SIRI has a certain value for the early diagnosis and prognostic assessment of elderly SPM patients.

## 1. Introduction

Pneumonia is a prevalent infectious disease of the respiratory system in elderly patients and an important cause of mortality [1]. Elderly patients suffer from low immune function, poor nutrition, and decreased respiratory resistance and are characterized by frequent underlying chronic diseases, such as cardiopulmonary diseases [2]. If the symptoms are atypical or the diagnosis is not standardized and timely, pneumonia is easy to progress to severe pneumonia (SPM) in elderly patients, which dramatically increases morbidity and mortality rates [3]. Some biological indicators of the human body have the characteristics of easy and rapid measurement, objectivity, and dynamic monitoring [4]. Therefore, searching for potential indicators that are effective in elderly patients with SPM has a critical clinical value for early disease prediction and prognosis judgment.

The Acute Physiology and Chronic Health Evaluation (APACHE‐II) score is a commonly used method for evaluating critical illness and prognosis, which can better reflect the damage to the respiratory system, blood system, liver and kidney function, and circulatory system when patients develop a disease but lacks the assessment of the degree of inflammatory response [5]. Relevant literature suggests that Gram‐negative bacillus infections or co‐infections can lead to elevated procalcitonin (PCT) levels in SPM patients, while C‐reactive protein (CRP) has been shown to increase with the exacerbation of SPM [6, 7]. In addition, leukocytes, neutrophils, and neutrophil/lymphocyte ratio can be used as predictors for outcomes of SPM patients [8, 9]. These findings confirm the significance of inflammatory responses in SPM. However, since the conditions of elderly SPM patients are complex, the indicators such as APACHE II score, CRP, and PCT levels alone cannot accurately assess the conditions of patients.

Systemic inflammatory response index (SIRI) comprises the absolute number of neutrophils, monocytes, and lymphocytes, with more significant prognostic value [10]. COVID‐19 patients with higher SIRI showed significantly lower survival [11]. SIRI is a potential biomarker of lung inflammation and serves as an independent risk factor for interstitial lung disease, pulmonary fibrosis, and lung cancer [12–14]. Importantly, SIRI may assist in predicting pneumonia in patients with cerebral hemorrhage and acute ischemic stroke [15, 16]. SIRI levels on postoperative days 1 and 3 are higher in SPM patients [17]. However, there are few studies on SIRI related to disease progression in SPM patients. Therefore, this study analyzed the correlation of SIRI with SPM and its value in the early diagnosis and prognostic assessment of elderly SPM patients.

## 2. Materials and Methods

### 2.1. Sample Size Estimation

The sample size was estimated using G∗Power 3.0.10 software developed by the University of Düsseldorf, with the following parameters: power = 0.95, *α* = 0.05, effect size = 0.5, and number of groups = 2. The minimal sample size required for the study was estimated to be 212. Considering a sample size attrition rate of approximately 20%, 266 patients were eventually included in the study.

### 2.2. Participants

This study retrospectively included 266 elderly patients with pneumonia admitted to our emergency department from August 2021 to January 2024. Clinical baseline and pathological data were pooled and analyzed, such as age, gender, history of smoking, history of alcohol consumption, history of chronic cardiopulmonary disease, history of diabetes, history of hypertension, BMI, neutrophil count, monocyte count, lymphocyte count, APACHE‐II score within 24 h of admission, PCT, and CRP. As previously described [18–20], patients within 24 h of admission were categorized into the SPM group (APACHE‐II score ≥ 20, *N* = 141) and the N‐SPM group (APACHE‐II score < 20, *N* = 125) according to the APACHE‐II scoring criteria. The study was performed in accordance with the *Declaration of Helsinki* and approved by the Ethics Committee of Shanghai Construction Group Hospital. All patients and their families volunteered to participate in this study (approval number: JGEC2024‐004).

### 2.3. Inclusion and Exclusion Criteria

Inclusion criteria for participants were as follows: (1) participants matching the diagnostic criteria of severe community‐acquired pneumonia in the American Thoracic Society and Infectious Diseases Society of America guidelines for the diagnosis and treatment of community‐acquired pneumonia in adults [21]; all enrolled patients followed the “Chinese Guidelines for the Diagnosis and Treatment of Community‐Acquired Pneumonia in Adults” [22], and the antibiotic therapy was initiated in the emergency department. (2) Participants with age ≥ 60 years; (3) participants who had good compliance and cooperated with the treatment and evaluation throughout the process. Exclusion criteria for participants were as follows: (1) participants combined with malignant tumors of the lungs or other parts; (2) participants combined with tuberculosis, noninfectious interstitial lung lesions, and other diseases; (3) participants with serious cardiovascular and cerebrovascular diseases; (4) participants with long‐term use of steroidal or nonsteroidal anti‐inflammatory drugs; (5) participants with psychiatric disorders that prevented normal communication; and (6) patients with incomplete information.

### 2.4. Calculation of Cytokines and SIRI

Elderly patients with SPM often present with dehydration and stress‐induced leukocytosis upon emergency admission. At this time, neutrophil and monocyte counts may be influenced by noninfectious factors (e.g., adrenaline release leading to the release of neutrophils from the marginal pool) [23]. Hence, all patients had blood drawn in the morning on an empty stomach to avoid interference from diet, circadian rhythms, blood concentration effects, and antibiotic interference windows.

On the second day of hospitalization, 5 mL of fasting venous blood was drawn from all patients, and the serum was separated by centrifugation at 4000 r/min (a centrifugation radius of 8 cm) for 20 min, with the supernatant placed at −20°C. CRP and PCT levels were measured with a fully automatic biochemical analyzer (Modular PPI, Roche, Switzerland). All experiments were carried out strictly under the instructions. SIRI was the value derived from peripheral blood neutrophils (N) × monocytes (M)/lymphocytes (L) [24].

### 2.5. APACHE II Score

The worst values among the relevant physiological indicators within 24 h of admission and the history of previous chronic diseases of the patients were subjected to APACHE II scoring. Higher scores indicated more severe conditions of the disease [20].

### 2.6. Follow‐Up

The survival status and postoperative condition of elderly SPM patients were recorded at 28 days after discharge through outpatient review or telephone follow‐up. SPM patients were assigned to either death (*N* = 76) or survival (*N* = 65) group based on their survival status. No patients were lost to follow‐up.

### 2.7. Statistical Methods

SPSS21.0 (IBM, Armonk, NY, USA) was used for data analyses. The Kolmogorov–Smirnov test was used to test for normal distribution. Normally distributed measurement data, which were summarized as mean ± standard deviation, were compared with the independent samples *t*‐test between two groups. Non‐normally distributed measurement data, which were presented as the median (minimum–maximum), were compared with the Mann–Whitney test between two groups. Count data were displayed as the number of cases (percentages), and the chi‐square test was utilized. The relationship between SIRI with APACHE II scores, CRP, and PCT was analyzed with Spearman correlation analysis. ROC curves were plotted to analyze the value of SIRI for the early diagnosis and short‐term prognostic assessment of SPM patients. Kaplan–Meier curves were utilized to analyze the relationship between SIRI and 28‐day survival of SPM patients. COX regression analysis was adopted to analyze risk factors for short‐term death of elderly SPM patients. *p* values were obtained from two‐sided tests, and the difference was considered statistically significant at *p* < 0.05.

## 3. Results

### 3.1. Comparison of General Information of Elderly SPM Patients and Non‐SPM Controls

A total of 266 elderly patients with pneumonia were included. No statistical difference was found between the two groups in age, gender, history of chronic cardiopulmonary disease, history of diabetes, history of hypertension, and BMI (all *p* > 0.05, Table 1). Importantly, the SPM group showed a higher percentage of patients with history of smoking and history of alcohol consumption compared to the N‐SPM group, accompanied by higher APACHE‐II scores, PCT, CRP, WBC, neutrophils, monocytes, and NLR and lower lymphocyte count (all *p* < 0.05, Table 1).

**Table 1:** Comparison of general data of elderly patients with severe and nonsevere pneumonia.

	SPM (*N* = 141)	N‐SPM (*N* = 125)	z/t/x^*2*^	*P*
Age (years)	71 (60–88)	70 (60–86)	0.230	0.818
Gender (n, %)			0.264	0.623
Male	61 (43.26)	58 (46.40)		
Female	80 (56.74)	67 (53.60)		
History of smoking (n, %)			5.454	0.022
Yes	50 (35.46)	28 (22.40)		
No	91 (64.54)	97 (77.60)		
History of alcohol consumption (n, %)			5.967	0.019
Yes	47 (33.33)	25 (20.00)		
No	94 (66.67)	100 (80.00)		
History of chronic cardiopulmonary disease (n, %)			0.122	0.750
Yes	26 (18.44)	21 (16.80)		
No	115 (81.56)	104 (83.20)		
History of diabetes (n, %)			0.545	0.498
Yes	43 (30.50)	33 (26.40)		
No	98 (69.50)	92 (73.60)		
History of hypertension (n, %)			0.285	0.685
Yes	13 (9.22)	14 (11.20)		
No	128 (90.78)	111 (88.80)		
BMI (kg/m^2^)	21.58 ± 1.86	21.53 ± 1.51	0.261	0.794
APACHE II (points)	27 (20–45)	17 (9–19)	14.112	< 0.001
CRP (mg/L)	18.02 (9.86–32.39)	11.48 (4.08–16.82)	13.048	< 0.001
PCT (ng/mL)	0.43 (0.05–1.74)	0.20 (0.05–0.36)	9.529	< 0.001
WBC (10^9^/L)	8.90 (2.80–23.19)	7.36 (4.55–10.14)	8.875	< 0.001
Neutrophil (10^9^/L)	6.65 (3.50–8.90)	5.92 (3.24–8.49)	4.162	< 0.001
Lymphocyte (10^9^/L)	1.76 ± 0.34	1.80 ± 0.32	1.098	< 0.001
Monocyte (10^9^/L)	0.65 ± 0.11	0.45 ± 0.12	14.633	< 0.001
NLR	2.27 (0.76–7.51)	1.50 (0.70–3.92)	9.561	< 0.001

*Note:* The Kolmogorov–Smirnov test was used to test for normal distribution. Normally distributed measurement data were expressed as mean ± standard deviation, and the independent samples *t*‐test was used for comparisons between the two groups. Nonnormally distributed measurement data were expressed as median (minimum−maximum), and the Mann–Whitney test was used for comparisons between two groups. Count data were expressed as number of cases and percentages, and the chi‐square test was used. APACHE II, Acute Physiology and Chronic Health Score; N‐SPM, nonsevere pneumonia; PCT, procalcitonin; SPM, severe pneumonia.

Abbreviations: BMI, body mass index; CRP, C‐reactive protein; NLR, neutrophil‐to‐lymphocyte ratio.

### 3.2. High SIRI Level in Elderly SPM Patients and Its Correlation With APACHE II Scores, CRP, and PCT

SIRI was higher in the SPM group [2.56 (0.65−5.45)] than in the N‐SPM group [1.42 (0.45−3.78)] (*p* < 0.001) (Figure 1(a)). According to the results of Spearman correlation analysis, SIRI levels in SPM patients were positively linked to APACHE II scores (*r* = 0.542, *p* < 0.001) (Figure 1(b)), CRP (*r* = 0.494, *p* < 0.001) (Figure 1(c)), and PCT (*r* = 0.568, *p* < 0.001) (Figure 1(d)).

Figure 1High level of SIRI in elderly SPM patients and its positive correlation with APACHE II scores, CRP, and PCT. Note: (a) SIRI levels in SPM and N‐SPM group; (b) correlation analysis between SIRI levels and APACHE II scores; (c) correlation analysis between SIRI and CRP levels; (d) correlation analysis between SIRI and PCT levels. ^∗∗∗^ *p* < 0.001.

**Figure:**
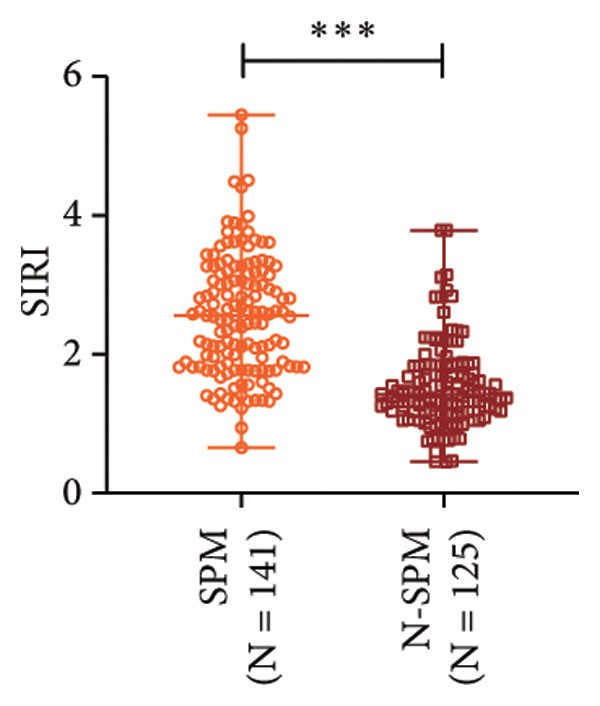
(a)

**Figure:**
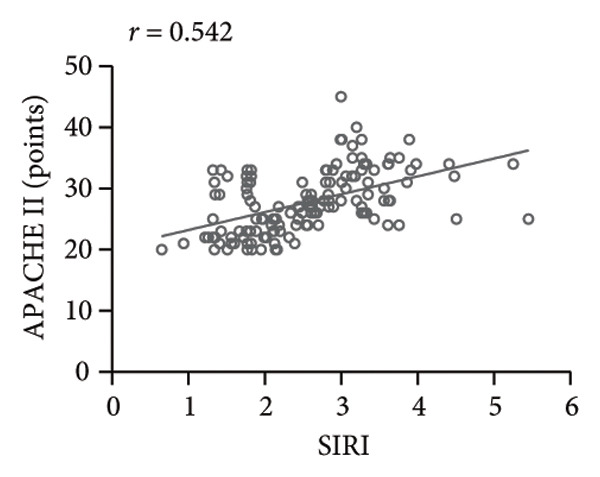
(b)

**Figure:**
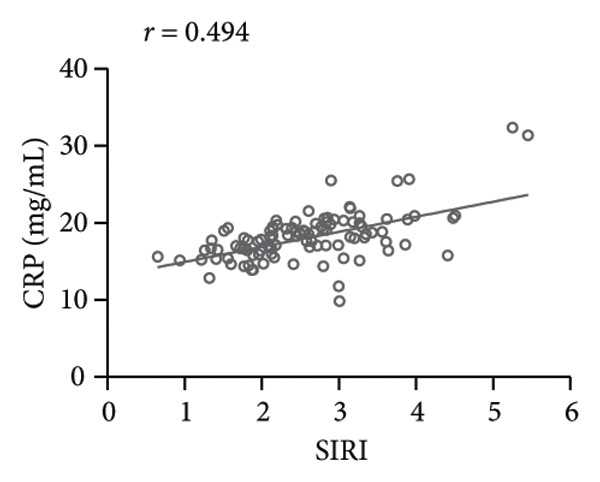
(c)

**Figure:**
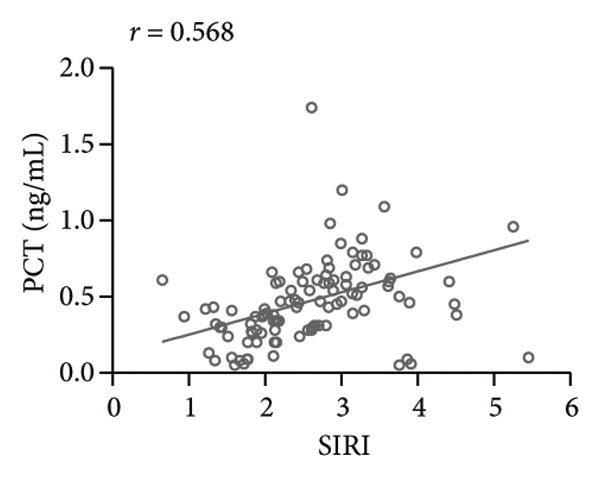
(d)

### 3.3. Diagnostic Efficacy of SIRI in Elderly SPM Patients

**Figure 2:**
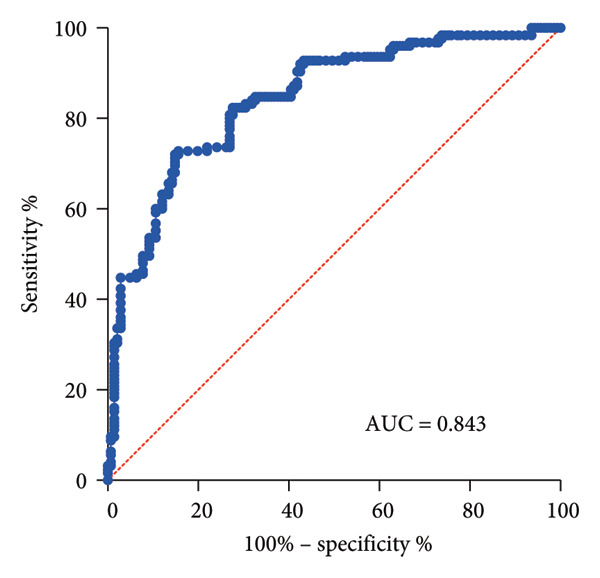
Analysis of the diagnostic efficacy of SIRI in elderly SPM patients. Note: A ROC curve was used to analyze the value of SIRI expression for the early diagnosis of SPM in the elderly.

ROC curve analysis displayed that the area under the curve (AUC) of SIRI in identifying elderly SPM patients was 0.843 (sensitivity, 84.40%; specificity, 72.80%; cut‐off value, 1.72) (*p* < 0.001) (Figure 2).

### 3.4. High SIRI Expression May Assist in Predicting the Short‐term Death of Elderly SPM Patients

Elderly SPM patients were assigned into death (*N* = 76) and survival (*N* = 65) groups based on 28‐day mortality of patients. SIRI levels were higher in the death group [3.00 (1.32−5.45)] than in the survival group [2.11 (0.65−3.91)] (*p* < 0.001) (Figure 3(a)). In the present study, the AUC of SIRI for predicting short‐term death in SPM patients was 0.806 (sensitivity, 76.32%; specificity, 84.62%; cut‐off value, 1.53). Additionally, the AUC of NLR was 0.835 (sensitivity, 75.00%; specificity, 90.77%; cut‐off value, 2.61). These data illustrated that SIRI and NLR had comparable predictive values for short‐term death, with no significant differences (*p* > 0.05) (Figure 3(b)). Altogether, high SIRI expression can assist in predicting short‐term death in SPM patients.

Figure 3High expression of SIRI aids in predicting short‐term death in elderly SPM patients. Note: (a) Differences in SIRI levels between death and survival groups; (b) ROC analysis of SIRI and NLR. ^∗∗∗^ *p* < 0.001.

**Figure:**
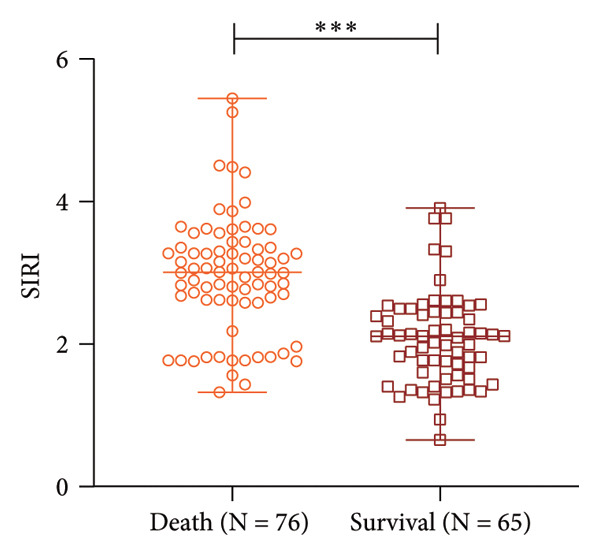
(a)

**Figure:**
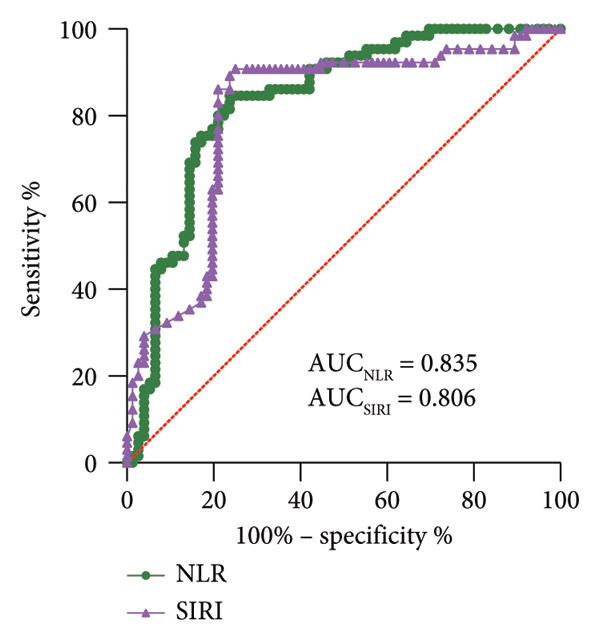
(b)

### 3.5. High SIRI Indicates the Increased Risk of Short‐term Death in Elderly SPM Patients

**Figure 4:**
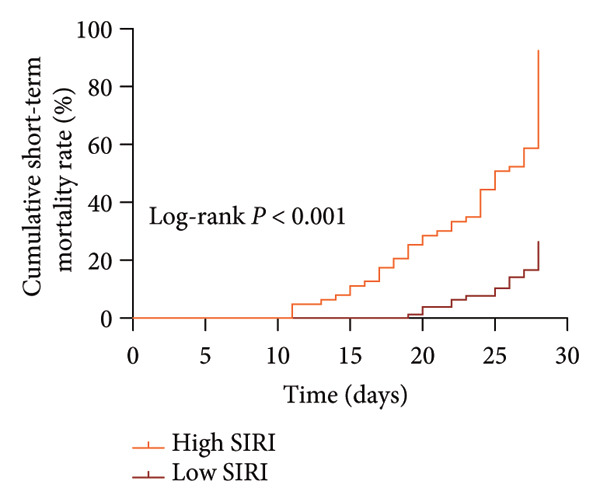
Kaplan–Meier survival curves to analyze the effect of SIRI expression on the short‐term survival of elderly SPM patients (log‐rank test).

Subsequently, elderly SPM patients were classified into high SIRI (SIRI > 2.61, *N* = 63) and low SIRI (SIRI ≤ 2.61, *N* = 78) groups according to the cut‐off value of 2.61. There were 57 deaths in the high SIRI group and 19 deaths in the low SIRI group (x^2^ = 141.00, *p* < 0.001). Then, the impact of SIRI expression on the short‐term survival of elderly SPM patients was analyzed with the Kaplan–Meier survival curve. Patients with high SIRI levels exhibited marked elevations in 28‐day mortality as compared to patients with low SIRI levels (log‐rank *p* < 0.001) (Figure 4).

### 3.6. SIRI Is a Potential Risk Factor for 28‐Day Mortality in Elderly SPM Patients

After adjustment for confounding factors (excluding SIRI‐related indexes such as neutrophil count, monocyte count, lymphocyte count, and NLR) and multivariate linear analysis (Table S1), variables including APACHE‐II scores, CRP, PCT, and SIRI were used for the multivariate Cox regression analysis to determine the influencing factors for 28‐day mortality in elderly SPM patients. The results unveiled that APACHE II scores (HR = 1.125) and SIRI (HR = 1.977) were independent risk factors for the short‐term death of elderly SPM patients (all *p* < 0.05) (Table 2).

**Table 2:** Screening for factors influencing short‐term death in elderly patients with SPM by logistical risk analysis.

	SE	Walds	*P*	HR	95% CI
APACHE II (points)	0.030	15.857	< 0.001	1.125	1.062–1.193
CRP (mg/L)	0.034	2.395	0.122	0.949	0.888–1.104
PCT (ng/mL)	0.425	1.454	0.228	1.670	0.726–3.843
SIRI	0.186	13.368	< 0.001	1.977	1.372–2.849

## 4. Discussion

Numerous risk factors associated with aging contribute to the heightened vulnerability of the elderly population to bacterial pneumonia, resulting in a substantial elevation of both morbidity and mortality rates [2]. Age‐associated cellular senescence can contribute to an upregulation of proinflammatory cytokines, heightened bacterial adhesion within the aged lung, and an increased vulnerability to pneumonia [25], highlighting the significance of inflammatory cytokines in pneumonia. SIRI denotes a balance between inflammatory responses and immune status and is a good predictor of pneumonia in patients with acute ischemic stroke [16]. This research illustrated elevated SIRI levels in elderly SPM patients and the certain value of SIRI for early diagnosis and prognostic assessment of elderly SPM patients.

Several publications have revealed the diagnostic capability of SIRI for pneumonia. For instance, Eissa et al. demonstrated that SIRI > 0.8 was essential in the diagnosis of COVID‐19 [26]. Compared with other inflammatory markers, SIRI may be a better predictor of SPM in patients with cerebral hemorrhage [17]. CRP and PCT are the most utilized biomarkers for pneumonia [27]. CRP > 30 mg/L and PCT > 0.25 ng/mL (90% sensitivity, < 25% specificity) could predict community‐acquired pneumonia in children [28]. CRP and PCT levels are related to the severity of chronic obstructive pulmonary disease complicated with pneumonia [29]. To further evaluate the diagnostic performance of SIRI for SPM, the present study firstly analyzed SIRI levels in SPM patients. Different from CURB‐65, PSI, or ATS/IDSA scores, which are commonly used in the clinical assessment of pneumonia severity [30–32], the APACHE‐II score encompasses three dimensions of acute physiological parameters, age, and chronic health status [33] and integrates age, acute multiorgan dysfunction, and chronic comorbidity burden, thereby more precisely reflecting disease progression in elderly patients with pneumonia. As previously described [18–20], most studies have used the APACHE‐II score for patient grouping. Therefore, the APACHE‐II score holds superior prognostic stratification capabilities in elderly pneumonia patients combined with multisystem diseases. As a result, the APACHE‐II score was utilized for patient grouping in the present study, which focuses on elderly patients with pneumonia who generally have a history of underlying diseases. Specifically, patients within 24 h of the current admission were categorized into the SPM group (APACHE‐II score ≥ 20) and the N‐SPM group (APACHE‐II score < 20) according to the APACHE‐II scoring criteria. Our results evinced that SIRI levels were higher in the SPM group than in the N‐SPM group and positively correlated with APACHE II scores, CRP, and PCT. Although this study only conducted a correlation analysis between SIRI and APACHE scores, it did not assess the correlation of other scores used for pneumonia patients, such as CURB‐65, PSI, or SMART‐COP scores, which may limit the applicability of the results. Similarly, SIRI was positively correlated with CRP in patients with gouty arthritis, and both SIRI and CRR had high diagnostic efficacy for gouty arthritis [34]. SIRI may be a promising predictor for all‐cause mortality in the elderly with heart failure and statistically positively correlated with CRP [35]. Serum PCT and CRP are independent predictors of bacterial pneumonia [36]. The ROC curve analysis in our study further showed that the AUC of SIRI in identifying SPM elderly patients was 0.843, indicating that high SIRI expression has an auxiliary diagnostic value for early diagnosis of elderly SPM patients, and further clinical assessment and other validated biomarkers (such as PCT and CRP) should be combined for comprehensive judgment. Likewise, SIRI had a higher predictive power for stroke‐related pneumonia in patients with cerebral hemorrhage [15]. SIRI could predict severe COVID‐19 and a low survival rate during hospitalization [37]. SIRI may be a better indicator than WBC and NLR in identifying postoperative pneumonia following meningioma resection [38]. Our research further provided evidence for the better prediction performance of SIRI for SPM. SIRI may be a simple way for the clinician to predict high‐risk populations of SPM before the symptoms appear.

NLR is a reliable predictor for postoperative pneumonia in elderly patients with hip fractures [39]. A former study unveiled that NLR could be used as a predictor of outcomes of SPM [9]. Intriguingly, the present study unraveled that SIRI and NLR were closely associated with short‐term death in elderly SPM patients, and their auxiliary diagnostic values were similar (SIRI: AUC, 0.806; sensitivity, 76.32%; specificity, 84.62%; cut‐off value, 1.53; NLR: AUC, 0.835; sensitivity, 75.00%; specificity, 90.77%; cut‐off value, 2.61). Furthermore, our data disclosed that SIRI was higher in the death group. Additionally, high expression of SIRI elevated the risk of short‐term death in elderly SPM patients. APACHE‐II scores and SIRI were independent risk factors for the short‐term death in elderly SPM patients. High SIRI was a strong predictor of invasive mechanical ventilation and mortality in COVID‐19 patients [40]. SIRI is a prognostic factor for worse long‐term outcomes after off‐pump coronary artery bypass grafting in patients with chronic coronary syndrome, and a preoperative value of SIRI > 1.27 indicates higher long‐term mortality risk [41]. Higher SIRI is independently associated with the risk of in‐hospital death for patients with infective endocarditis [42]. Adults with SIRI levels of > 1.43 had higher risk of all‐cause and cardiovascular death [43], and this correlation was significant among patients with hypertension [44] and the obese population [45]. Overall, SIRI, comprising three inflammatory biomarkers, has certain auxiliary predictive value for the short‐term death of elderly SPM patients. Notably, although SIRI demonstrated favorable discriminatory ability for early diagnosis and short‐term mortality in elderly SPM patients (1.72, 1.53), it cannot be used independently for diagnosis or prognosis prediction. Furthermore, its cut‐off value may vary depending on population differences. Whether it is applicable to elderly SPM patients in different regions, countries, or those with concomitant hematological diseases, immunosuppressive therapy, or viral infections requires further validation.

This study had several limitations. First, the number of cases of elderly SPM patients in our study is relatively limited, which calls for multicenter studies with large sample sizes to further validate our findings. Second, this study only analyzed the correlation of SIRI with CRP, PCT, and APACHE II scores and did not analyze the correlation of SIRI with neutrophils and lymphocytes, which may lead to different SIRI values at the time of onset in elderly SPM patients. Hence, the correlation of SIRI with neutrophils and lymphocytes in elderly SPM patients should be analyzed.

Conclusively, this study unraveled that SIRI was highly expressed in elderly SPM patients and was positively correlated with CRP, PCT, and APACHE II scores. Additionally, high SIRI levels had certain value for the early diagnosis and prognostic assessment of SPM in elderly patients, increased the 28‐day mortality rate, and was an independent risk factor for short‐term death in elderly SPM patients. Our findings provide a reference for the application of SIRI to the early diagnosis and prognostic assessment of SPM in elderly patients.

## Ethics Statement

The study was in accordance with the *Declaration of Helsinki* and approved by the Ethics Committee of Shanghai Jiangong Hospital. All patients and their families volunteered to participate in this study (approval number: JGEC2024‐004).

## Consent

The authors have nothing to report.

## Disclosure

All authors read and approved the final manuscript.

## Conflicts of Interest

The authors declare no conflicts of interest.

## Author Contributions

X.X. is the guarantor of integrity of the entire study; H.X. and X.X. contributed to the definition of intellectual content, study design, and statistical analysis; M.L. contributed to the study concept, literature research, and manuscript review; S.Y. and H.C. contributed to the clinical studies, data analysis, and manuscript editing; H.X. and S.Y. contributed to the data acquisition and manuscript preparation.

## Funding

This work was supported by the Scientific Research Project of Shanghai Municipal Health and Family Planning Commission (funding no. 201840263).

## Supporting Information

Table S1 Multivariate linear analysis of APACHE II, CRP, PCT, and SIRI.

## Supporting information


**Supporting Information** Additional supporting information can be found online in the Supporting Information section.

## Data Availability

The datasets used and analyzed during the current study are available from the corresponding author on reasonable request.
