# Supplementary material for: Systemic Inflammatory Response Index Has a Value for the Early Diagnosis and Short‐Term Prognostic Assessment of Severe Pneumonia in the Elderly
Source: Can Respir J. 2026 Jan 6;2026:6968736. doi: 10.1155/carj/6968736 (PMC12774799; doi:10.1155/carj/6968736)
Supplement: Supplementary file 1 — Supporting Information Additional supporting information can be found online in the Supporting Information section. [file CARJ-2026-6968736-s001.docx]

**Table S1** Multivariate linear analysis

|  | Tolerance | variance inflation factor (VIF) |
| --- | --- | --- |
| APACHE II (points) | 0.704 | 1.421 |
| CRP (mg/L) | 0.644 | 1.552 |
| PCT (ng/mL) | 0.771 | 1.296 |
| SIRI | 0.444 | 2.255 |
